# Supplementary material for: Patterns of Intron Gain and Loss in Fungi
Source: PLoS Biol. 2004 Nov 30;2(12):e422. doi: 10.1371/journal.pbio.0020422 (PMC532390; doi:10.1371/journal.pbio.0020422)
Supplement: Table S1 — Also available at http://genes.mit.edu/NielsenEtAl/. (4.3 MB ZIP). [file pbio.0020422.st001.zip › NielsenEtAl/html/1411.html]

AN6695.1.NCU05267.1.MG06063.1.FG01948.1


```
 CLUSTAL W (1.82) Multiple Sequence Alignments - Introns Inserted


Sequence 1: NCU05267.1	1181 aa
Sequence 2: FG01948.1	1142 aa
Sequence 3: MG06063.1	1138 aa
Sequence 4: AN6695.1	1170 aa
Alignment Length: 1227 aa
Number Identitical Residues: 562 aa
Alignment Score (without introns) 28126


MG06063.1 	MDRARKR1E----L~RALNDRAWTGE---~---------------------KS1LIAINS
NCU05267.1	MDRQRRR1E----L~RALNSRAWNGE---~---------------------KD1LFPVSK
FG01948.1 	MDRARKR1E----L~RTLNEKAWDGESGM~NHLIYPYPTTSPRPRQLTQTITD~VFPVGK
AN6695.1  	MTKSKHL~NSGGPV1QRLNCSILQGK-GS1EFITAGIATLQPGGKRMDRQRKH1IFPVSK
          	* : ::  ::...: : **     *: .  .      .: ..         .  ::.:..

MG06063.1 	SLDSSLRKNTSFMKRLRTAISAANLNTFIQEIKTLSLHKYLSEIISACYEGLCRLKTPSE
NCU05267.1	SLDSTLKKNTAFIKRLRTTISAATQSTFLQEIRTVSLSKYLSEIISACYEGLCKLKSPGD
FG01948.1 	TLDSSLKKNTAFIKRLRTAVTAATLSTFLQEIRTLSLHKYLSEIISACYEGLCRLKSPGE
AN6695.1  	SLDSSLKKNTAFIKRLRTGINAAAQQTLLTDIRTLSLHKYLSEIISACYEGLCKLKSPGE
          	:***:*:***:*:***** :.**  .*:: :*:*:** ***************:**:*.:

MG06063.1 	IEAGVEILSALHQRFGPDEFTQHLGWLLGRGMATPEKSYLKTLPPETREKEERERLVRQR
NCU05267.1	IEAGVEIVSALHQRFGPADFTEHLGWLLGKGMATPDKSFLKTLTPEAREKEEKDRLVRQR
FG01948.1 	IEAGVEIVSALHQRFGPSEFTEYLAWLLGKGMATPDKGILKSLAPEVREKEEKERIIRQR
AN6695.1  	ITAGVEITSALHQRFGPAEFTRQIGWLLGRGLSSPDKSQLKTLSQELREREEKERLSRHR
          	* ***** ********* :**. :.****:*:::*:*. **:*. * **:**::*: *:*

MG06063.1 	VLIRVVTELWLVGVLRTLDDTTRPDDG-TKGVPGKPQETKARTS-------IKGGGAEPF
NCU05267.1	VLLRVVTELWLVDVLRTLDDIARPDDA-TRPTTGARAEPKPRSSS------GKGGGAEPF
FG01948.1 	ALLRVVTELWLVGVIRTLDDVSKPDDA-TKGAAGKTTELKTRTS--------KASGAEPF
AN6695.1  	VLLKVVTELWLVGVLRTLDDVERPEDVGSKGKDGAGLGNKVVDGVSRNRNPAGDKEPEPF
          	.*::********.*:*****  :*:* .::   *     *   . : . ..     .***

MG06063.1 	PLEVLKDLLGHDREHVNLPLLVTFVKSFSWDVFGIKAAGADGRKTVEEDGATKTANGGTD
NCU05267.1	PLEVLKDLLNHDRDHANLPLLVIFVKAFSWDILGIKAAGQDGRKTVEEDGATKGAEETEG
FG01948.1 	PLEVLKDLLGHDREHANLPLLVIFVKSFSWDILGVKPTGADGRKTVEEDGSTEGHEAEQG
AN6695.1  	PLEVLKDLLGHDRDHSNLPLALLFAKSFSWDVLGAKAAE-EGRKTVEADGATAMPAETQN
          	*********.***:* **** : *.*:****::* *.:  :****** **:*       .

MG06063.1 	LDHQELHDHGGTGDDEPLTPAELQERFRNVLKRYFDDVKAHLERDQKAISNQSRRNAEAY
NCU05267.1	DGSEDKEEGAGADQDGPFAPPELQERFKNILKRYFEDVKGHLVRDQKAIYNQSRKNAEAY
FG01948.1 	D---DGENGTSSDEDQPFAEPEMRDRFRNILKKYFDDVKGHIIRDQKSIQSQARRNAEAY
AN6695.1  	G------DEATAEDDTPLAPEKIQVRFKSILNRYLEDVKAHVMRDQKALTAQSRRNAEAY
          	       :   : :* *::  ::: **:.:*::*::***.*: ****::  *:*:*****

MG06063.1 	VKSGEVFEDRQANFDKQVKAQERLVSNAQVLADMLGAEMPDLKDDTDPMANANASIGLVR
NCU05267.1	VKSGEVFEDRQSNFEKQVKAQERLVANAQVIADIIGAEMPELKDTDEGNAAN-GTIGLVK
FG01948.1 	VKSGEVFEDRQANYEKQVKAQERLVSNAQVVADAIGVEMPDLKDSDDSLAASNGSIGLVK
AN6695.1  	VKSGEIFEDRQANFERQTKSLEKLVANTQVLCEILGVEMPALATQEASDPAASGGIGLVK
          	*****:*****:*:::*.*: *:**:*:**:.: :*.*** *       .  .. ****:

MG06063.1 	TGEYLRGQGEGAGIWEDEEERRFYENLVDLKGKVPAILLEDAKKKKAETDEQVGKR----
NCU05267.1	AGEYLRGQGEGPGIWEDEEERRFYENLVDLKGKVPGILLEEVKKKKAD-DEQVGRK----
FG01948.1 	TGDYLRSMGDGAGIWEDDDERRFYENLVDLKGKVPAILLEDGKKKKTDTDEQVGKK----
AN6695.1  	TSEYLRGQGEGPGIWEDEEERRFYENLVDLKGKVPAVLLDDGKKKKTETGEPTKKKESEA
          	:.:***. *:*.*****::****************.:**:: ****:::.* . ::.:.:

MG06063.1 	----VDTPETTDIAKAAES--ADDQSTAIANKTTGAQVDAILLRLPDMITKDNVDQTAID
NCU05267.1	----IDPVEAAEAAKAAEAAAADDQSIAIANKTIGAQVDALLVRLPELISKDAIDQMAID
FG01948.1 	----LDPAEVPETTKIAET--ADDQSTSIANRTIGAQVDALLARLPELTNKDIVDQTAID
AN6695.1  	GHESSEKTEPQSQISEERTTEADDQSMAIASKTVGAQVDALLAKLPELQTKEQVDQMALD
          	. .: :  *  .  .  .:: ***** :**.:* ******:* :**:: .*: :** *:*

MG06063.1 	FCFLNSKASRNRLVKALTDVPKGRSDLLPFWARLVATLGQHMPDIPKGVVEYLDAEFRSL
NCU05267.1	FCFLNSKASRNRLIKALTDVPKGRTDLLPNWARLVATLGQYMPDIPKGLVDYLDNEFRSL
FG01948.1 	FCFLNSKASRNRLVKALTEVPKGRSDLLPSWSRLVATLGRYMPDVPKGLVDYLDAEFRSL
AN6695.1  	FCFLNSKASRNRLVKAISDIPKGRIDLLPLYSRLVATLGQYMPDIPQGLTTYLDEEFRSL
          	*************:**::::**** **** ::*******::***:*:*:. *** *****

MG06063.1 	QRRKDKDFLSQVRLSNIRYLAELTKFNIVPEHVVFHCLKVSLEDFSKMNIEVICNLLENC
NCU05267.1	QRRKEKEFLGQVRLSNIRYLAELTKFGIVPEHVVFHCLKVSLDDFSRMNIEIICNLLENC
FG01948.1 	QRRKEKDFLGQVRLSNIRYLAELTKFGIVPEHVVFHCLKVSLDDFSRMNIEILCNLIENC
AN6695.1  	QRRKSKEFLGQVRMSNIRYLAELTKFGVVPEHIIFHCFKVSLDDFSRMNIEIIGHLLENC
          	****.*:**.***:************.:****::***:****:***:****:: :*:***

MG06063.1 	GRYLLRNPETSPRMMSFLDTLQRKKSVQHIGQAEKMLIENAVYYVNPPERPAIEQKERTP
NCU05267.1	GRYLLRNPETSPRMASFLETLQRKKSVQHIGQAERMLIENAVYYVDPPQRPAIEVKERTP
FG01948.1 	GRYLLRNPETAPRMMSFLETLQRKKSVQHIGQPERMLIDNAVYYVDPPERSAIEQKERTP
AN6695.1  	GRYLLRNPDTSPRMASFLETLGRKKTVQHLGPQERMIIENAVYYVDPPQRPAIQQKERTP
          	********:*:*** ***:** ***:***:*  *:*:*:******:**:*.**: *****

MG06063.1 	MELFVRKLVYVDLSKRNYSKVLKQIRRLHWEEKE~VVAILTKVFTKPGRVKYGNIYLLAI
NCU05267.1	MELFIRKLIYGDLTKRNYSKILRHIRRLHWEEKD~VVAILHKVFSKPGKVKYGNIHLLAI
FG01948.1 	MELFVRKLIYVDMTKRNYSKILKQIRRLHWEEAE0VVAILEKVFSKPGKVKYGSVHLLGI
AN6695.1  	MESYIRKLIYLDMNKRNYTKILKSVRKLHWEEPE~VVHILERVFSKPAKVKYGNIHLLAI
          	** ::***:* *:.****:*:*: :*:***** : ** ** :**:**.:****.::**.*

MG06063.1 	LLGAICKYHVVFTVTVIDTLVESITFGLEQNEFKFNQRRIAEAKYLGELYNYRMFDHSVI
NCU05267.1	LLSALYKYHGEFVVGVIDQVTESICLGLESNDFKFNQRRIAEVKYLGELYNYRMLEHPVI
FG01948.1 	LLSALYRYHPAFVVKVIDNVIESMTFGLEQNDYRFYQRRIAEVKYLGELYNYRMLEHPVI
AN6695.1  	LVSALYRYHQGFVIGIVDNILEYITLGLEQNDFKFNQKRIAEVKYLGELYNYKMIDSPVI
          	*:.*: :**  *.: ::* : * : :***.*:::* *:****.*********:*:: .**

MG06063.1 	FDTMYKILTFGHG~G-PPVPGRINPFDLSDDFFRIRLIATILETCGMFFNKGVQGKKLDY
NCU05267.1	FDTMYKIMTFGHG~G-PPVPGRVNPFDLPDDFFRIRLISTILETCGMFFNKGAAGKKLDF
FG01948.1 	FDTMYKIMTFGYG1G-PPVPGKYSPLDPPDDFFRIRLVSTMLETCGMFFNRGAAGKKLDY
AN6695.1  	FDTLYRIVTYGHE1GGTPMPGKINVLDMPDDFFRIRLVCQLLDTCGHCFDRGSAKKKLDF
          	***:*:*:*:*:  *..*:**: . :* .********:. :*:***  *::*   ****:

MG06063.1 	FLSFFQ0YYIFTKEPLPMDIEFIVQDTYALTRPQWKLATNLEEASRAFQMAIAQDQKNSG
NCU05267.1	FLSFFQ~YYIYTKNPLPMDVEFLVQDIFALTRPQWKLASNLEEASKVFQLAMVQDQKTSG
FG01948.1 	FLSFFQ0YYIFTKASLPMDIEFLVHDTFALTRPQWKIASGLEEAVKAFQLAVAQDQKSAG
AN6695.1  	FLKFFQ0YYICTKDPLPMDIDFLVQDTYSLTRPQWTLVTELDEASRIFGEAVAQNFKPQ-
          	**.*** *** ** .****::*:*:* ::******.:.: *:** : *  *:.*: *   

MG06063.1 	LDKVLEQDDGTSGPSSDDGEGDGDDLNIADVDDDEDVESDED~D~DHDG-SHVDSDSEDE
NCU05267.1	VERVVEPEDNDSDVSDDEDGMVPDEDEDDESDDDDGGEAEDN~E~QQDNTSRYNSESEEE
FG01948.1 	VDKGVDVDDATSDALSDDD-----NDEDGDDNDDSASEEEEV~E0DMDEDSDSDSAFDEE
AN6695.1  	-EEKPEPEEESEDSGSDED---LEEDAFPEADEEGESSDEAD0V~SPNAERNDDSESEEE
          	 :.  : ::  ..  .*:.    .:    : :::   . :     . :     :*  ::*

MG06063.1 	AIVVTREEEEVNPEDEAEFEREYAKMMAESLDSRKFDRKPLFDVPLPMRTKN--------
NCU05267.1	EIVVTRQQEEVDPEEEAEFEREFAKMMAESLDSRKFERKQQFDLPLPIKLKPRESTAVGE
FG01948.1 	AIVVTRQEEEVDPEDEADFEREYAKMMAESLESRKFERKQLFDVPLPVRSKTREATST--
AN6695.1  	QIFVTRQEEERDPEAEAEFDREFEKMMAESVESRKFERKAVFDIPLPMRRAARDPTAEVT
          	 *.***::** :** **:*:**: ******::****:**  **:***::    ..::   

MG06063.1 	-----QVGEGPVTNAPGTMAFSLLTKRGNRQQ0TRTVELPSDSNFAIAMKNQQAAEREEQ
NCU05267.1	SSAANEAGPSEAPPSGEKMAFSLLTKKGNRQQ0TRTVELPSDSHFAIAMKKKQQAEKEEQ
FG01948.1 	-----EGGEGESPP-GHTMAFSLLTKRGNRQQ0TRTVELPSNSTFAVAMKNQQQAEREEQ
AN6695.1  	AANSTPAPVSSPAQSSGTMAFSLMTKKGNKQQ~TRTIDLPSDSTFAVAMRSQQQADREEQ
          	:: :.    .  . :  .*****:**:**:** ***::***:* **:**:.:* *::***

MG06063.1 	QRIKNLVLNLDLGENETEDG1ND~NKPTSYHHNHG------------------NRQG~RD
NCU05267.1	QRIKNLVLNYDLRENDDYEG~NE~KLNAHHHNRSDLRLNKERGGQRVRKLQLSDVDC2PN
FG01948.1 	QRIKNLVLNYDLQQNEDPDG~GT~DPQLPQLYRN--------------------INI~HN
AN6695.1  	QRIKNLVLNYEMS-NETDST~EE1PVEKRSPARVD-------------------KSG~TN
          	********* ::  *:  .             . .                    .   :

MG06063.1 	RNAQRGR----------KLQLSDVDW2LVVASTHARETPRG
NCU05267.1	RTEPLGAGAGVWEEVNYKRATDFSGR~EILEFASVGLSGQG
FG01948.1 	RNQ----------------ATNDQRR~IITTV---------
AN6695.1  	RSAFRSR----------KLQLSDVNW~--------------
          	*.   .           .   .
```
